# Rapid development of extensive myocardial calcification in a diabetic patient with multi-organ failure: a case report

**DOI:** 10.1093/ehjcr/ytag146

**Published:** 2026-03-03

**Authors:** Sami Marzouki, Jochen Juré, Michiel Nachtergaele, Daniel Devos

**Affiliations:** Department of Cardiovascular Radiology, Ghent University Hospital, Corneel Heymanslaan 10, Ghent 9000, Belgium; Department of Cardiology, Ghent University Hospital, Corneel Heymanslaan 10, Ghent 9000, Belgium; Department of Cardiology, Ghent University Hospital, Corneel Heymanslaan 10, Ghent 9000, Belgium; Department of Cardiovascular Radiology, Ghent University Hospital, Corneel Heymanslaan 10, Ghent 9000, Belgium

**Keywords:** Myocardial calcification, Dystrophic calcification, Diabetic ketoacidosis, Critical illness, Multi-organ failure, Case report

## Abstract

**Background:**

Extensive myocardial calcification is a rare phenomenon typically associated with severe systemic illness. It may develop rapidly in critically ill patients following myocardial injury, particularly in the context of sepsis, vasoplegia, and profound metabolic derangements.

**Case Summary:**

A 39-year-old man with poorly controlled type 1 diabetes mellitus presented in a comatose state due to severe diabetic ketoacidosis and hypothermia. Laboratory investigations revealed acute kidney injury, hypophosphataemia, and an elevated inflammatory profile. Initial imaging, including non-contrast CT, was unremarkable. During his ICU stay, he developed multi-organ failure with vasoplegia requiring high-dose vasopressors and transient cardiac dysfunction. On Day 14, he experienced an in-hospital cardiac arrest. Post-resuscitation CT unexpectedly revealed extensive calcification of the left ventricular myocardium, which had not been present on earlier imaging. Despite temporary haemodynamic stability, he suffered irreversible neurological injury and died on Day 19. Autopsy confirmed diffuse dystrophic myocardial calcifications without evidence of infarction or fibrosis.

**Discussion:**

This case illustrates rapid-onset dystrophic myocardial calcification occurring in the setting of severe metabolic and inflammatory stress. Prolonged vasopressor exposure, acidosis, and systemic inflammation likely contributed to myocardial injury and subsequent calcium deposition. Awareness of this rare complication may improve diagnostic vigilance and support early prognostic discussions in similar patients.

Learning points

- Rapid-onset dystrophic myocardial calcification can occur in critically ill patients with severe metabolic and inflammatory stress, such as diabetic ketoacidosis and vasoplegic shock.
- CT imaging is essential for detecting myocardial calcifications, which may be missed on echocardiography and can impact prognosis and management.

## Introduction

Myocardial calcifications can arise from various aetiologies. Dystrophic calcification develops in previously damaged myocardium, whereas metastatic calcification reflects systemic derangement of calcium-phosphate homeostasis.^1^ Extensive myocardial calcification is rare and has been most commonly reported in patients with sepsis or fulminant myocarditis.^2,3^ The pathophysiology of such calcification is poorly understood and is thought to result from myocardial inflammation and calcium dysregulation.^1–4^ Prognosis in these patients is poor, with mortality rates reported up to 33%, and no established treatment guidelines are currently available.^2,3^ We present the case of a 39-year-old man with multi-organ failure secondary to diabetic ketoacidosis (DKA) who rapidly developed extensive myocardial calcification.

## Summary figure

**Figure:**
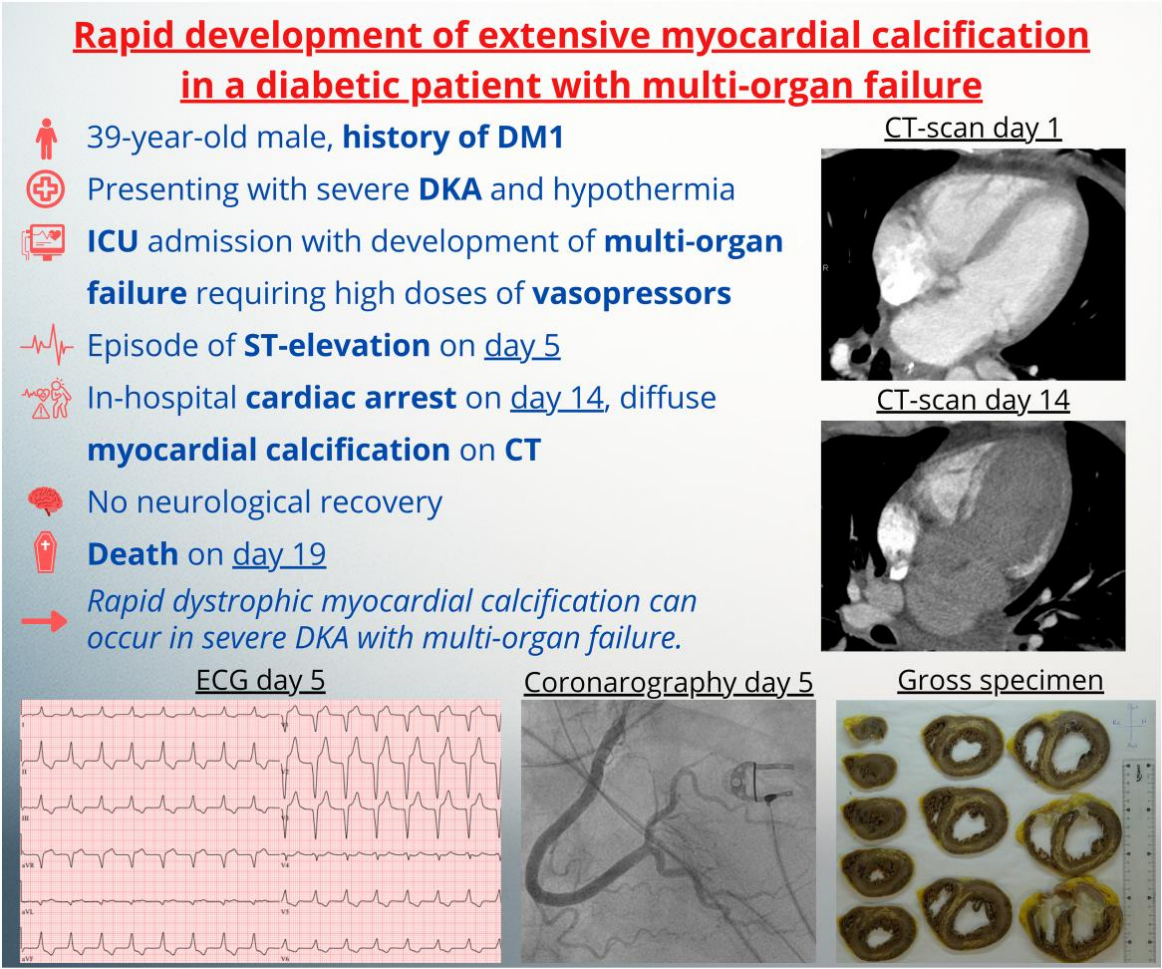


## Case presentation

A 39-year-old male patient with a history of poorly managed type 1 diabetes mellitus, Graves’ disease, and chronic alcohol use was found in a comatose state. On arrival to the emergency department, he was haemodynamically unstable with a heart rate of 46 bpm and a blood pressure of 96/54 mmHg. The patient had normal lung sounds, and abdominal examination was unremarkable. There were no signs of external trauma, except for a small wound on the right upper leg, without any sign of inflammation. He was also severely hyperglycaemic (929 mg/dl), in profound metabolic acidosis (pH 6.78, pCO_2_ 36.6 mmHg, bicarbonate 5.3 mmol/l, and lactate 1.26 mmol/l) and profoundly hypothermic (core temperature of 26°C). Urine dipstick was positive for ketonuria, confirming the diagnosis of DKA. Laboratory testing revealed a leukocytopenia (white blood cell count 0.72 × 10^9^/L), acute kidney injury with a creatinine level of 2.3 mg/dl and normal sodium and potassium levels (144 mmol/l and 3.8 mmol/l). C-reactive protein was elevated (130 mg/l), and there was a hypophosphataemia of 0.21 mmol/L with a normal serum calcium of 2.32 mmol/L. Aggressive fluid resuscitation and insulin therapy were initiated. A non-contrast head and thoraco-abdominal computed tomography (CT) revealed no abnormalities. The patient was admitted to the ICU, where empirical broad-spectrum antibiotics were initiated for presumed infection in the context of severe vasoplegia and escalating vasopressor requirements. However, despite extensive evaluation, no infectious source was identified. Blood cultures, urine cultures, and cultures from nasotracheal aspiration all remained negative. Urinalysis showed no bacteriuria, no nitrites, and no leukocyturia. Bacterial and viral respiratory swabs were negative. Careful clinical inspection revealed no skin or soft tissue infection, including no ulcers or diabetic foot lesions. Following multidisciplinary discussion, and in the absence of microbiological, clinical, or radiological evidence of infection, empirical broad-spectrum antibiotics were discontinued after 48 h.

**Figure 1:**
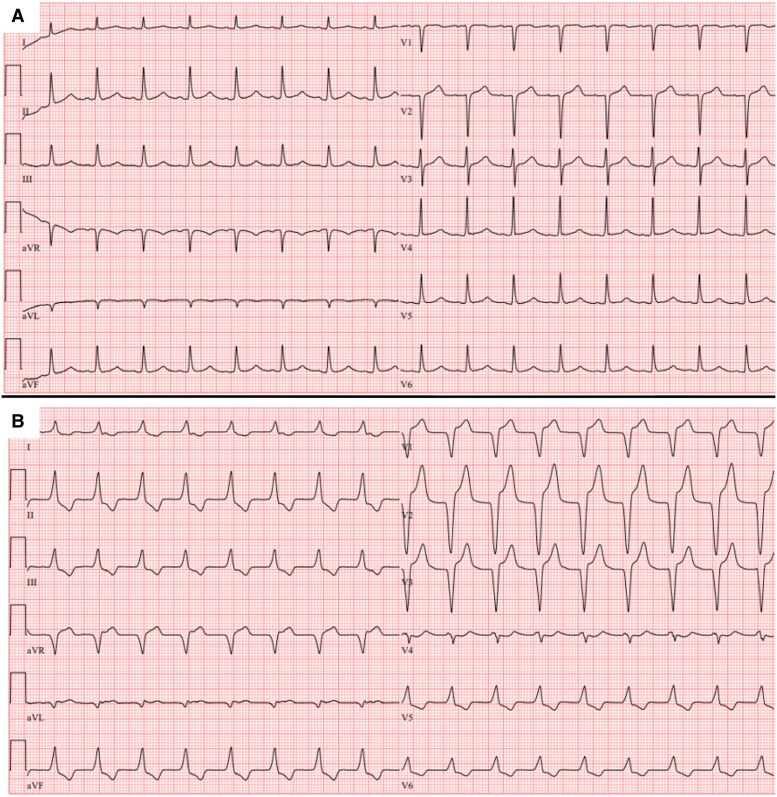
Baseline ECG showing no abnormalities (*A*). ECG obtained 5 days later demonstrates marked ST-elevation in the anterior leads and T-wave inversion in the inferior and lateral leads (*B*).

**Figure 2:**
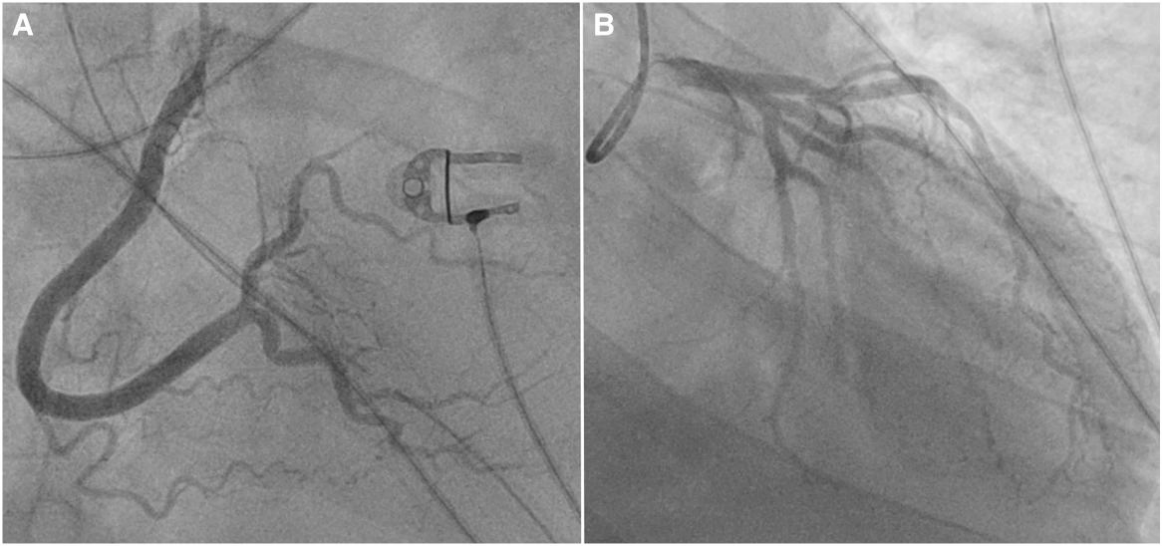
Coronary angiography on Day 5 showed no significant coronary artery disease.

During his ICU stay, he developed multi-organ failure, including worsening renal failure requiring temporary renal replacement therapy, hepatic failure, and pancytopenia. On Day 5 of admission, his ECG changed markedly from baseline and was compatible with an anterior ST-elevation myocardial infarction (*Figure 1*), with elevated troponin levels (188 pg/ml and 325 pg/ml after 1 h, up to 4390 pg/ml following coronary angiography). Serum potassium level remained within normal range at that time (3.9 mmol/L), excluding hyperkalaemia as a cause of the observed conduction abnormalities. Echocardiography showed moderately decreased systolic function with no regional wall motion abnormalities. Given these findings, an urgent coronary angiogram was performed, but showed no significant coronary artery disease (*Figure 2*).

**Figure 3:**
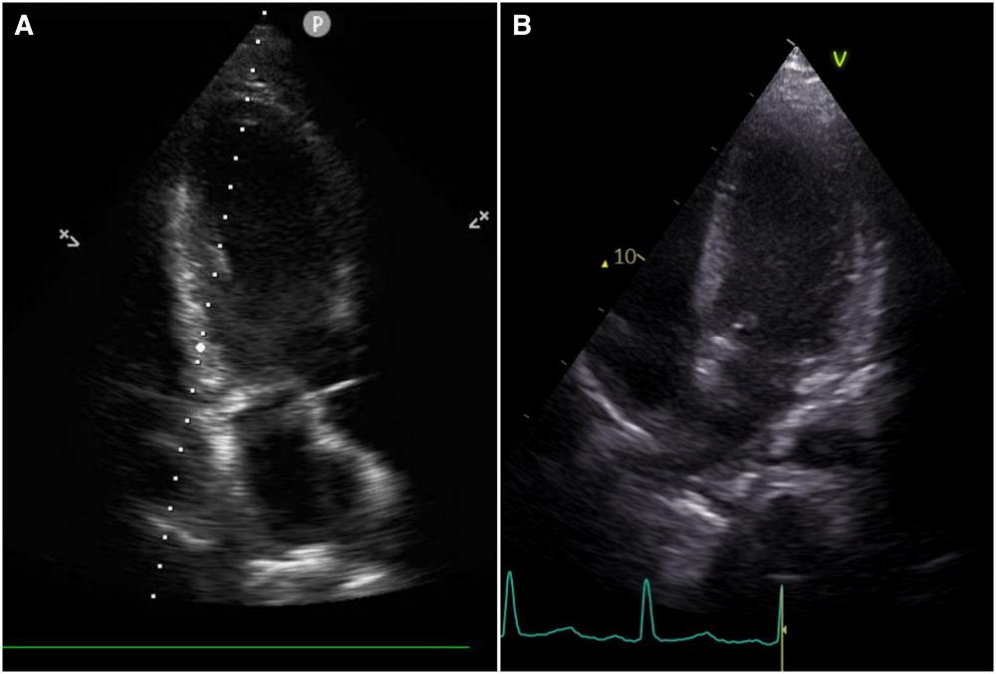
Echocardiography demonstrating hyperechogenicity of the myocardium (*A*), compared with normal myocardial echogenicity on an earlier examination (*B*).

**Figure 4:**
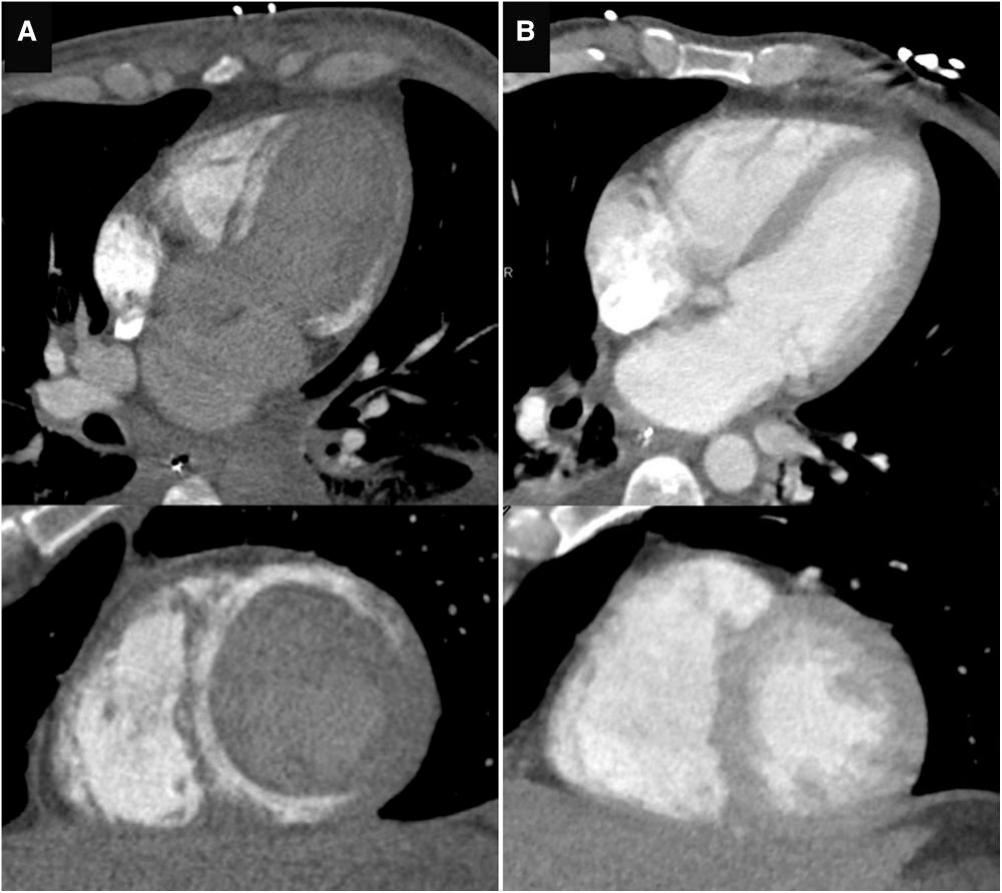
Multiplanar reformats of a chest CT after in-hospital cardiac arrest showing diffuse myocardial calcifications (*A*), compared with the normal chest CT performed 14 days earlier (*B*).

The patient’s condition initially improved, including his neurological status, and he could be transferred to the endocrinology ward 12 days post-admission. However, 2 days later, he suffered a sudden in-hospital cardiac arrest. Despite prompt and successful resuscitation, he sustained extensive hypoxic-ischaemic brain injury. A follow-up echocardiogram showed a normalized systolic function, though with a newly observed hyperechoic appearance of the myocardium (*Figure 3*). NT-proBNP at that time was 4258 pg/ml. A contrast-enhanced chest CT was performed to rule out pulmonary embolism and instead revealed diffuse calcifications in the left ventricular myocardium that were not present on the earlier CT (*Figure 4*).

The patient was readmitted to the ICU, where he developed a short episode of ventricular fibrillation but otherwise remained haemodynamically stable. However, there was no recovery of neurological function, and multimodal neuroprognostication suggested a dismal prognosis. As such, a decision was made to discontinue life-supporting therapy. The patient eventually died 19 days after admission.

**Figure 5:**
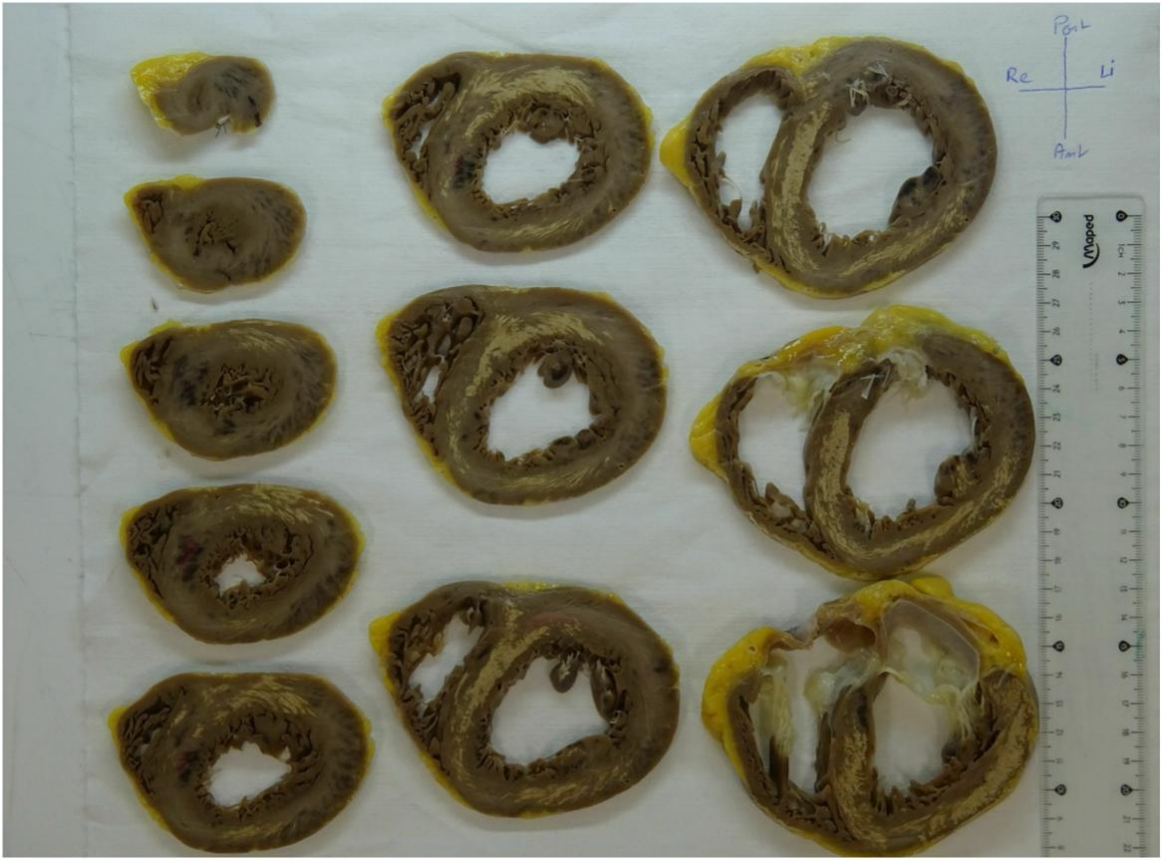
Gross specimen from autopsy showing extensive whitish discolouration of the left ventricular myocardium, consistent with calcific infiltration.

An autopsy confirmed the diffuse myocardial calcifications (*Figure 5*). Histologic examination indicated dystrophic calcifications, associated with localized tissue damage rather than systemic calcium abnormalities, but no evidence of myocardial ischaemic injury or fibrosis.

## Discussion

Rapidly developing extensive myocardial calcification is rare, documented only in isolated case reports or small series. A recent systematic review identified merely 75 cases over the past decade, underscoring its exceptional rarity.^2^ While a broad range of aetiologies, including hepatic or renal failure, myocardial infarction, and post-heart transplantation states have been described, sepsis and fulminant myocarditis remain the most frequently reported triggers, accounting for approximately half of these cases.^2,3^ In our case, although he presented with a septic shock-like clinical picture, no infectious aetiology was identified. This suggests that the profound metabolic and inflammatory stress associated with DKA and multi-organ failure may have been sufficient to induce myocardial injury and subsequent dystrophic calcification. Although a minor or subclinical infection cannot be entirely excluded, the absence of microbiological, clinical, and radiological evidence supports severe DKA and associated metabolic stress as the predominant driver in this case.

Pathophysiologically, myocardial calcification can be categorized as either dystrophic or metastatic. Dystrophic calcification results from localized myocardial injury or necrosis and typically occurs in patients with normal calcium-phosphate metabolism. In contrast, metastatic calcification stems from systemic disturbances in calcium homeostasis, most commonly seen in chronic kidney disease, where sustained hypercalcemia or hyperphosphatemia promotes calcium deposition.^1^ In critically ill patients, the rapid development of myocardial calcification strongly supports a dystrophic mechanism. Severe systemic inflammation and metabolic acidosis, frequently observed in sepsis and DKA, create a milieu in which extensive myocyte injury leads to rapid calcium deposition within days to weeks, far more rapidly than in chronic or healed myocardial injuries such as myocardial infarction.^2,4,5^

While diabetes itself has not been directly linked to myocardial calcification, acute hyperglycaemic crises such as DKA, especially when complicated by systemic inflammation or sepsis, can represent a severe myocardial insult through mechanisms including severe acidosis, accumulation of ketone bodies, oxidative stress, and electrolyte disturbances such as hypophosphataemia and transient calcium imbalance.^5–8^ Another important, though often under-recognized contributor is prolonged high-dose catecholamine therapy. Beta-adrenergic stimulation augments *trans*-sarcolemmal calcium influx, raises ATP demand, and exacerbates oxidative stress, culminating in myocyte necrosis. A clear dose-duration signal between adrenergic load and calcium deposition was demonstrated earlier.^4,9^ The combination of severe DKA, multi-organ failure, and sustained vasopressor therapy likely acted synergistically to amplify cellular injury and accelerate dystrophic calcification.^4–6,10^

Clinicians should remain vigilant for myocardial calcification as a potential complication in critically ill patients presenting with refractory cardiac dysfunction, particularly those with multi-organ failure or prolonged vasopressor dependence. In addition, myocardial calcifications may serve as an arrhythmogenic substrate, potentially leading to life-threatening arrhythmias, as was likely the case in our patient.^1,2,11^ Early recognition and prompt correction of underlying metabolic disturbances may mitigate the progression and impact of myocardial calcification.

Diagnostic identification of myocardial calcification typically occurs incidentally via imaging. While historically discovered at autopsy, CT now serves as the gold standard for detection, demonstrating patchy or ring-like myocardial calcium deposits with high sensitivity.^2,3,12^ Echocardiography may identify myocardial echogenicity changes, although subtle early calcifications can be overlooked, underscoring the necessity of CT for definitive diagnosis.^3^ Cardiac MRI complements the diagnostic workup through identification of myocardial scar via late gadolinium enhancement, although it lacks specificity for calcium detection without CT correlation.^11^

No specific treatments are available to reverse myocardial calcification; thus, management primarily involves addressing underlying causes and providing supportive care. Meticulous correction of acid-base and calcium-phosphate abnormalities and judicious use of catecholamines is advisable. Survival outcomes are variable and closely linked to the severity of the initiating illness. While historical outcomes were dismal, recent advances in critical care have improved survival.^2^ Nonetheless, survivors often face significant long-term morbidity, including restrictive or dilated cardiomyopathy and arrhythmic risks, highlighting the need for prolonged cardiac follow-up.^3,11^

In summary, our case highlights extensive myocardial calcification as an important yet infrequently recognized complication in critically ill patients experiencing severe metabolic and inflammatory stress. Clinicians should be aware of this rare complication in metabolically unstable ICU patients, where early detection may help guide prognosis and care decisions.

## Data Availability

The data underlying this article are available in the article and in its online supplementary material.

## Lead author biography

Dr. Sami Marzouki is a final-year radiology resident at Ghent University Hospital with a strong interest in cardiovascular and interventional radiology.

## Notes

**consent:** The authors confirm that written consent for submission and publication of this case report, including images and associated text, has been obtained from the patient’s next of kin in line with COPE guidance.
